# Characterization and Nanoparticle Immobilization of a *Thermotogaceae* Esterase Active on Aromatic and Aliphatic Esters

**DOI:** 10.1021/acsomega.6c05063

**Published:** 2026-10-07

**Authors:** Pietra Bruna Barbosa Bento, Dnane Vieira Almeida, Iara Ciancaglini, Ellen K.B. Roman, João Renato Carvalho Muniz, Roberta Albino dos Reis, Fabio Marcio Squina, Wanius Garcia

**Affiliations:** † Centro de Ciências Naturais e Humanas, 74362 Universidade Federal do ABC (UFABC), Santo André, São Paulo CEP 09210-170, Brazil; ‡ Laboratory of Molecular Sciences, University of Sorocaba (UNISO), Sorocaba, São Paulo CEP 18060-501, Brazil; § São Carlos Institute of Physics (IFSC), University of São Paulo (USP), São Carlos, São Paulo CEP 13563-590, Brazil

## Abstract

Esterases are a class of enzymes that catalyze the hydrolysis of carboxylic ester bonds and are widely utilized in the food, pharmaceutical, and detergent industries. In this study, we report the identification and comprehensive characterization of a novel esterase (TbEst) from the *Thermotogaceae* family, a group of thermophilic bacteria. The predicted three-dimensional structure of TbEst displays a classical α/β-hydrolase fold with a conserved Ser-His-Asp catalytic triad. TbEst was cloned, heterologously expressed, and purified via a multistep chromatographic protocol. The melting temperature of TbEst at pH 8.0 was 78 °C, confirming its thermostability. Furthermore, TbEst exhibited activity against p-nitrophenyl acetate, confirming its esterase activity, with optimal performance at pH 8.0 and 50 °C. Notably, TbEst exhibited catalytic activity toward broad aromatic and aliphatic esters, reinforcing its versatility for diverse biotechnological applications. Furthermore, the present study describes the successful nanoscale immobilization of TbEst onto Ag/AgCl nanoparticles (TbEst@Ag/AgCl-NPs) via a facile photochemical route. TbEst@Ag/AgCl-NPs were highly stable, with different geometries including spherical and cubic; it was crystalline in nature, and had a mean diameter of 100 nm. Notably, TbEst retained its catalytic activity upon nanoscale immobilization onto Ag/AgCl-NPs. Furthermore, nanoscale immobilization conferred enhanced thermostability and significantly prolonged the active lifetime of the bioconjugate compared to the free enzyme. The data described here should provide a useful basis for future research and biotechnological applications of esterases and nanoimmobilized esterases.

## Graphical abstract

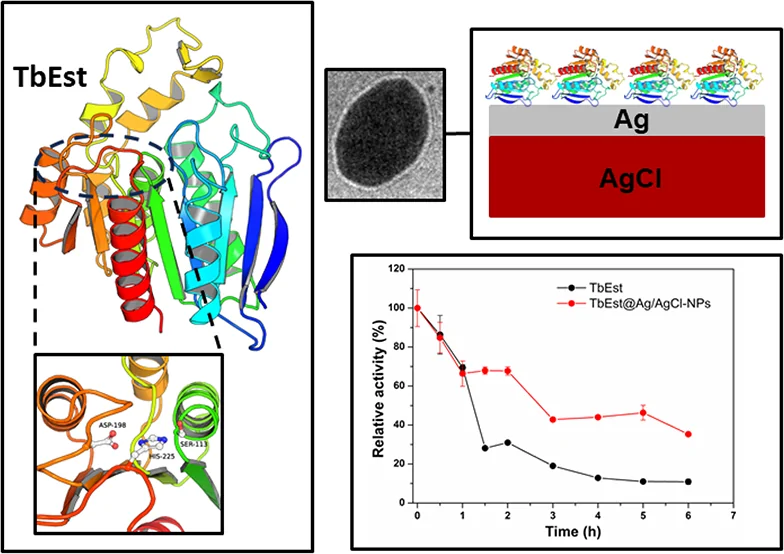

## 1 Introduction

Esterases (EC 3.1.1.1) are a diverse group of enzymes belonging to the hydrolase class, and specifically the α/β-hydrolase fold family that is found across a wide range of organisms, including bacteria,^1^ fungi,^2^ and plants.^3^ Their significant industrial potential stems primarily from their ability to catalyze the hydrolysis of ester bonds, efficiently converting esters into alcohol and carboxylic acid products.^4,5^ Esterases are remarkably versatile biocatalysts that extend far beyond simple hydrolysis to facilitate a diverse range of synthetic transformations, including esterification, transesterification, and various exchange reactions like acidolysis and aminolysis. The esterase active site contains a conserved catalytic triad composed of Ser-His-Asp amino acid residues. These residues work in concert to facilitate nucleophilic attack, reaction activation, stabilization, and proton transfer processes essential for catalysis.^6,7^

The rapid growth of enzyme-mediated processes in biochemical industries is driven by their superior sustainability, energy efficiency, and functional versatility across a wide range of temperatures.^8^ In an industrial context, esterases have been widely studied for their ability to degrade materials containing ester bonds.^9–11^ Furthermore, esterases are used in a variety of processes for the degradation of synthetic and natural polymers and for the synthesis of optically pure compounds.^12–14^ For example, esterases are employed to produce biofuels from lignocellulose plant biomass^15^ and to degrade agricultural pesticides.^16^ More recently, these enzymes have emerged as promising tools for the enzymatic hydrolysis of terephthalate esters found in PET plastics.^17–19^ Collectively, these applications not only help reduce environmental pollution but also improve the efficiency of industrial processes by promoting the conversion of materials to useful products and serving as essential tools in diverse biotechnological fields.^20^

Enzymes from thermophilic microorganisms offer solutions to the challenges of industrial biocatalysis due to their thermostability and activity at higher temperatures. Accordingly, several esterases from thermophilic bacteria have been identified.^21–28^ The family *Thermotogaceae*, part of the *Thermotogales* order and the *Thermotogae* phylum, consists largely of thermophilic organisms, with optimum growth at temperatures above 45 °C.^27^ The *Thermotogales* order is also quite unique due to its characteristic outer sheath, known as a “toga”. This “toga” creates a distinctive periplasmic space that is crucial for polysaccharide degradation. Enzymes derived from these microorganisms, including esterases, exhibit remarkable thermal stability, a characteristic that is highly advantageous for industrial processes that often operate under harsh conditions.^28^ Among the experimentally validated structures of *Thermotogaceae* esterases, the acetyl xylan esterase TM0077 (PDB ID: 3M81) and EstA (PDB ID: 3DOH), both from *Thermotoga maritima*, represent the highest-resolution models available.^29,30^

Nanoscale immobilization of enzymes such as esterases on nanoparticles (NPs) enhances their biocatalytic potential by improving stability and activity while enabling efficient separation and reuse in industrial processes.^31–35^ This technique consists of attaching or trapping enzymes to a solid support, such as a matrix or membrane.^36^ NPs offer an extensive surface area and a matrix that can protect proteins from adverse environmental conditions and facilitate the separation and recovery of enzymes after use. The nanoscale immobilization process is particularly relevant for esterases, as it enables more efficient and prolonged use of these enzymes in industrial processes. Additionally, nanoscale immobilization can improve the specificity and reaction rate of esterases, expanding their applications in various areas, from the cosmetics industry to bioremediation.^33–38^ Various nanoscale immobilization methods, including physical adsorption, covalent bonding, entrapment, and *in situ* biosynthesis, have been developed to optimize enzyme performance.^4^ In particular, biosynthesis methods employ microorganisms or biological molecules as nontoxic reducing, capping, and stabilizing agents.^33–39^

In this study, we present a comprehensive characterization of a novel esterase from a *Thermotogaceae* bacterium (TbEst) prospected from a public database. To gain deeper insight into its molecular structure, the TbEst three-dimensional structure was generated and evaluated using molecular modeling. TbEst was cloned, heterologously expressed, and purified using chromatographic methods. Our results indicate that TbEst is a thermostable enzyme that hydrolyzes aromatic and aliphatic esters, confirming its esterase activity. Furthermore, we report the nanoscale immobilization of TbEst onto Ag/AgCl-NPs via a photochemical synthesis method. This bioconjugation significantly enhances TbEst’s thermostability, extending its operational range to higher temperatures.

## 2 Materials and Methods

### 2.1 Materials and Chemicals

Reagents including Luria–Bertani (LB) medium, imidazole, isopropyl-β-D-thiogalactopyranoside (IPTG), kanamycin (kan), nickel-nitrilotriacetic acid resin (Ni-NTA), and all organic substrates (aromatic and aliphatic esters) were acquired from Merck. Protein molecular weight markers for SDS-polyacrylamide gel electrophoresis (SDS-PAGE) were acquired from Bio-Rad. The Superdex 75 10/300 GL column was purchased from GE Healthcare. All chemicals were of analytical grade.

### 2.2 Prospection of Encoding Sequences for Thermophilic Esterases

The *in silico* prospection of TbEst, a putative esterase belonging to the *Thermotogaceae* family, was performed via the UniProt database (https://www.uniprot.org/). The enzyme corresponds to the gene locus GX457_08790 and is documented under entries UPI000B065873 and A0A7 × 7W8F2. Enzyme candidates from thermophilic and hyperthermophilic microorganisms were selected by using taxonomy filters. Further, optimal growth temperatures for these strains were retrieved from the Bac*Dive* database (https://bacdive.dsmz.de/), a comprehensive repository containing information on thousands of bacterial and archaeal strains.^40^ To enhance the likelihood of obtaining a soluble enzyme, solubility predictions were carried out using the ProteinSol tool (https://protein-sol.manchester.ac.uk/). Subsequently, BLASTp searches against the Protein Data Bank (PDB, https://www.rcsb.org/) provided additional structural and functional context for the chosen enzyme candidates. TbEst consists of 249 amino acid residues as shown in Table S1, with a predicted molecular mass of 28.33 kDa.

### 2.3 Molecular Modeling Using the AphaFold3 Program

We predicted a structural model for TbEst (protein sequence containing 249 amino acid residues) employing the AlphaFold3 program.^41,42^ We performed the structural modeling using six recycles to ensure convergence and model quality. Model quality was assessed using the predicted local distance difference test (pLDDT) score from AlphaFold, with residues showing pLDDT > 90 considered highly confident. Additionally, evaluation of the final model using MolProbity and the Ramachandran plot confirmed good stereochemical quality. Structural superimposition of the TbEst model with known esterases confirmed the canonical α/β-hydrolase fold and the spatial arrangement of the Ser-His-Asp catalytic triad, with RMSD values indicating close structural similarity.

### 2.4 Recombinant Expression and Purification of TbEst

The cDNA encoding TbEst was codon-optimized, synthesized (GenScript USA Inc.), and cloned into the pET-28a­(+) vector. The expression and purification of TbEst were performed using previously described methodology with slight modifications.^17^ Briefly, BL21­(DE3) cells transformed with the expression plasmid were grown overnight in LB medium containing 50 μg/mL kan at 37 °C with shaking at 200 rpm. The overnight culture was inoculated into 1 L of LB medium containing 50 μg/mL kan and incubated at 37 °C and 200 rpm until an optical density of 0.6 at 600 nm was reached. Recombinant protein expression was induced with 0.5 mM IPTG, followed by incubation at 20 °C and 200 rpm for 16 h. Subsequently, the cells were harvested by centrifugation (6,000 × g, 10 min at 4 °C) and resuspended in 20 mM Tris-HCl buffer (pH 7.4) containing 20 mM NaCl. Following cell disruption by sonication, the supernatant was collected after centrifugation (6,000 × g, 30 min at 4 °C). The supernatant was then loaded onto a Ni-NTA affinity column and washed with a buffer consisting of 20 mM Tris-HCl (pH 7.5), 20 mM NaCl, and 5 mM imidazole. The bound TbEst was eluted with a buffer containing 20 mM Tris-HCl (pH 7.5), 20 mM NaCl, and 100 mM imidazole. Further purification of TbEst was performed via size-exclusion chromatography (SEC) on a Superdex 75 10/300 GL column (GE Healthcare) coupled with an ÄKTA purifier 10 system. Collected fractions were analyzed via SDS-PAGE for purity evaluation. TbEst concentration was quantified by measuring absorbance at 280 nm, utilizing a theoretical extinction coefficient derived from its amino acid composition (ε_280 nm_= 28,420 M^–1^cm^–1^).

### 2.5 Esterase Activity Assay

Biochemical characterization was performed to evaluate the effects of pH and temperature on TbEst activity, as well as its thermal stability, using previously described methods.^17^ One unit of enzyme activity (U) was defined as the amount of enzyme that catalyzes the conversion of 1 μmol of substrate per min. Specific activity is reported in U/mg. Esterase activity toward p-nitrophenyl acetate (pNPA), p-nitrophenyl butyrate (pNPB), p-nitrophenyl palmitate (pNPP), and p-nitrophenyl decanoate (pNPD) was assayed in a volume of 100 μL containing 50 mM buffer, 2 mM substrate, and 0.15 μg/mL (5.3 nM) TbEst. Reactions were incubated for 5 min, and the absorbance was measured at 415 nm. A blank control containing the substrate in buffer without the enzyme was systematically measured and subtracted from the total absorbance at every tested condition. The methodology described by Almeida et al.^17^ was used to evaluate the activity on the following substrates: diethyl phthalate, tripropionin, geranyl acetate, cyclohexyl butyrate, glyceryl tributyrate, octyl acetate, vinyl butyrate, vinyl benzoate, vinyl propionate, γ-valerolactone, butyl acetate, glyceryl triacetate, and phenyl propionate. To evaluate the optimal temperature, enzyme activity was measured using pNPA as the substrate in 50 mM Tris-HCl buffer (pH 8.0) over a temperature range of 30 to 80 °C. To evaluate the optimal pH, enzymatic activity was measured at 50 °C using pNPA as the substrate in 50 mM acetate buffer (pH 4.0–5.5), sodium phosphate buffer (pH 6.0–7.0), and Tris-HCl buffer (pH 7.5–9.0). The kinetic parameters *V*_max_ and *K*_m_ were determined from a Michaelis–Menten plot (velocity versus pNPA concentration). Experimental data were fitted to a rectangular hyperbola using nonlinear regression. The assays were performed in triplicate across three independent experiments.

### 2.6 Biophysical Characterization of Recombinant TbEst

Circular dichroism (CD) spectra were collected on a Jasco J-815 spectropolarimeter from 190 to 260 nm in a 0.1 cm quartz cuvette at 20 °C. An average of eight scans was recorded for each spectrum, with a scan rate of 100 nm/min, spectral bandwidth of 1 nm, and response time of 0.5 s. Spectra were collected using 0.25 mg/mL TbEst in 20 mM Tris-HCl buffer (pH 8.0), with appropriate buffer blanks subtracted prior to data analysis. Thermal denaturation of TbEst was monitored by measuring temperature-induced changes in ellipticity at 220 nm. The data were fitted using a nonlinear regression with the Boltzmann sigmoidal function to calculate the melting temperature (*T*_m_). Dynamic light scattering (DLS) measurements were performed on a Zetasizer Nano ZS system (Malvern Instruments Ltd., Malvern, UK) to determine the hydrodynamic radius (*R*_h_) of TbEst. Purified TbEst (1 mg/mL) in 20 mM Tris-HCl, pH 8.0, was analyzed in a quartz cuvette, and the corresponding molecular mass (kDa) was estimated using the Zetasizer software.

### 2.7 Photochemical Synthesis of Silver/Silver Chloride Nanoparticles Capped with TbEst

Photochemical synthesis of silver/silver chloride nanoparticles (Ag/AgCl-NPs) capped with TbEst (TbEst@Ag/AgCl-NPs) was performed using a previously described methodology with slight modifications.^33–35^ Briefly, a 10 mL solution of 1 mM Tris-HCl buffer (pH 8.0), 1 mM AgNO_3_ and 0.1 mg/mL TbEst was exposed to visible light (450–600 nm) for 1 h at room temperature under static conditions. Control experiments were performed in the absence of either AgNO_3_ or TbEst under identical conditions. The resulting TbEst@Ag/AgCl-NPs were purified by centrifugation and repeated washes (three steps) with ultrapure water to remove unbound TbEst.

### 2.8 Thermostability of Free TbEst and TbEst@Ag/AgCl-NPs

For thermostability analysis, both free TbEst and TbEst@Ag/AgCl-NPs were incubated at 60 °C for intervals ranging from 0 to 6 h. Following each incubation period, aliquots were withdrawn and assayed for residual activity using 2 mM pNPA in 20 mM Tris-HCl buffer (pH 8.0) at 60 °C for 5 min, followed by absorbance measurement at 415 nm. In the recycling experiments, TbEst@Ag/AgCl-NPs were recovered by centrifugation and resuspended in fresh substrate under optimal reaction conditions. All experiments were performed in triplicate, with free TbEst serving as the control.

### 2.9 Characterization of TbEst@Ag/AgCl-NPs Bioconjugate

The absorbance of the TbEst@Ag/AgCl-NPs was measured using a double-beam UV–vis spectrophotometer (Biachrom Libra) from 200 to 800 nm. Particle size and zeta potential of purified TbEst@Ag/AgCl-NPs in ultrapure water were evaluated by dynamic light scattering (DLS) and electrophoretic light scattering (ELS)^43^ at 25 °C using a Zetasizer Nano ZS (Malvern Instruments, UK) equipped with 10 mm path-length cuvettes. Fourier-transform infrared spectroscopy (FTIR) analyses of free TbEst (used as a control) and TbEst@Ag/AgCl-NPs were performed on a PerkinElmer instrument in transmittance mode from 4,000 cm^–1^ to 400 cm^–1^ with a resolution of 4 cm^–1^. Transmission electron microscopy (TEM) was performed on a Thermo Scientific Talos F200X G2 instrument operating at 200 kV. For sample preparation, a single drop of the TbEst@Ag/AgCl-NPs solution was deposited onto a carbon-coated copper grid and dried at room temperature. The particle size distribution was quantified by analyzing the TEM images with software ImageJ (National Institutes of Health—NIH). Energy-dispersive spectroscopy (EDS) was employed to determine the elemental composition of the TbEst@Ag/AgCl-NPs. X-ray diffraction (XRD) analysis was performed to investigate the crystalline structure of the TbEst@Ag/AgCl-NPs. Measurements were performed using a STADI-P powder diffractometer (Stoe, Darmstadt, Germany) equipped with a Mythen 1K silicon microstrip detector (Dectris, Baden, Switzerland). Diffraction patterns were obtained using Cu Kα_1_ radiation (λ = 1.54056 Å) under operating conditions of 40 kV and 40 mA. Data were collected over a 2θ range of 20° to 80°, with a step size of 0.015° and an integration time of 100 s for every 1.05° scanned.

## 3 Results and Discussion

### 3.1 Molecular Modeling of TbEst

To gain insights into the molecular structure of TbEst, a molecular model was generated by the AlphaFold3 server.^41,42^ The final predicted three-dimensional model for TbEst is shown in Figure 1. To validate this homology model, we employed the predicted template modeling (pTM) and interface predicted template modeling (ipTM) scores. The pTM score evaluates the overall accuracy of the predicted fold relative to the true structure, with scores above 0.5 suggesting a likely correct fold.^41,42^ The ipTM score, on the other hand, assesses the accuracy of predicted subunit relative positions within a complex, with values above 0.8 indicating high-confidence predictions. The AlphaFold3 model (Figure 1) represents the monomeric unit of TbEst. The predicted template modeling (pTM) score for the monomer was 0.96, indicating a high-confidence fold (pTM > 0.5). The model exhibited a mean pLDDT of 97.03, with 97.6% of residues in the “very high confidence” category (pLDDT > 90) and only 2.4% in the “good confidence” range (70–90). No residues were predicted with “low confidence” (pLDDT < 70), underscoring the exceptional structural reliability of the model. MolProbity analysis yielded a score of 1.54, and Ramachandran statistics showed 97.6% of residues in favored regions, 2.0% in allowed regions, and 0.4% as outliers, confirming the excellent stereochemical quality of the model.

**Figure 1:**
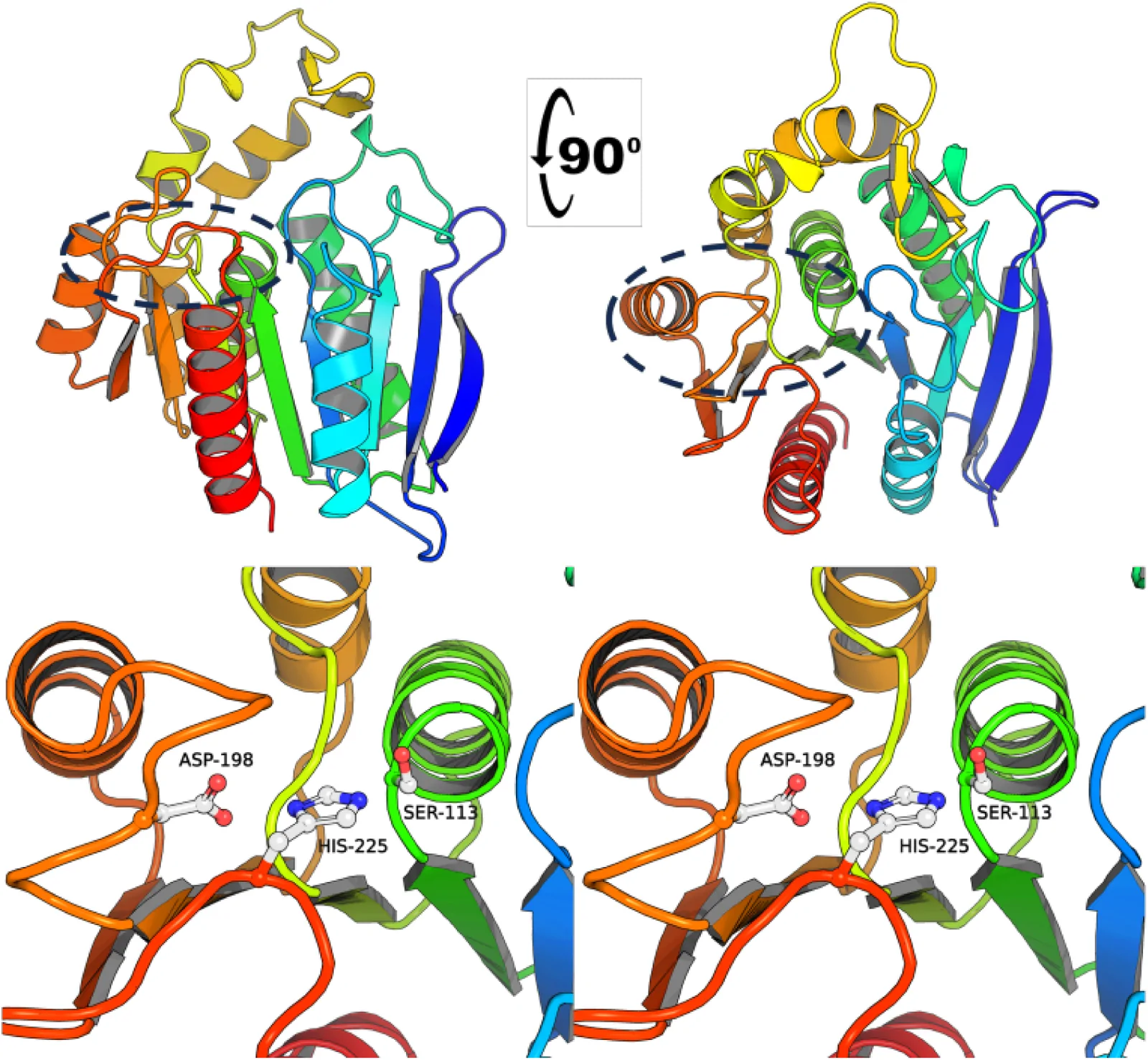
Molecular model of TbEst. The model is depicted in cartoon format, color-coded by amino acid sequence from dark blue at the N-terminal to red at the C-terminal. The stereo view location of the catalytic triad, consisting of residues Ser113, His225, and Asp198, is highlighted with a dashed-line ellipse in the upper part of the figure and represented in balls and sticks format in the lower part. The two images are rotated 5° relative to each other to enable 3D depth perception of the catalytic triad geometry. The images were created using PyMOL v3.0.2.

Structural superimposition with known esterases further validated the canonical α/β-hydrolase fold and the spatial arrangement of the catalytic triad (Figure S1). The molecular model obtained (Figure 1) exhibited a classical α/β-hydrolase fold, comprising a central core of eight β-sheet strands, one buried α-helix, and six solvent-exposed α-helices.

Sequence alignment revealed that TbEst shares approximately 34.9% sequence identity (and 49.8% similarity) with the acetyl xylan esterase TM0077 from *Thermotoga maritima* (PDB ID: 3M81),^29^ confirming that both enzymes belong to the same α/β-hydrolase family while exhibiting sequence divergence. While both enzymes adopt the canonical α/β-hydrolase fold, structural superposition with TM0077 and EstA from *T. maritima* (PDB ID: 3DOH)^30^ yielded RMSD values of 2.5 Å and 2.9 Å, respectively, confirming the conserved core while revealing differences in surface loop regions (Figure S1). EstA and TM0077 were selected for structural superimposition because they represent the highest-resolution, experimentally validated folds available for *Thermotogaceae* esterases.^29,30^

Furthermore, the location of the catalytic triad, consisting of amino acid residues Ser113, His225, and Asp198, is highlighted in Figure 1 and Figure S1. This triad participates in the hydrolysis of ester bonds. Ser acts as a nucleophile, attacking the carbonyl carbon of the substrate, while His facilitates the deprotonation of Ser, enhancing its nucleophilicity. Asp acts to stabilize the positive charge on His and promote a stable catalytic microenvironment.^17,44,45^ The amino acid residues sequence of TbEst also contains the conserved G-X-nucleophile-X-G-G motif (Gly111-Tyr112-Ser113-Met114-Gly115-Gly116), which is characteristic of the environment surrounding the catalytic nucleophile (Table S1). Recent research has also highlighted the importance of structural elements surrounding the catalytic triad, such as disulfide bonds, which can influence the stability and activity of enzymes.^46^ The catalytic triad not only defines the catalytic mechanism but also contributes to the substrate specificity and overall efficiency of esterases, making it a focal point in the study of enzyme engineering and biocatalysis.^47,48^

### 3.2 Expression and Purification of Recombinant TbEst

Recombinant TbEst was successfully expressed in *E. coli* and primarily localized to the soluble fraction following cell lysis and centrifugation. A two-step purification protocol, involving affinity and size-exclusion chromatography, was used to purify recombinant TbEst (Figure 2).

**Figure 2:**
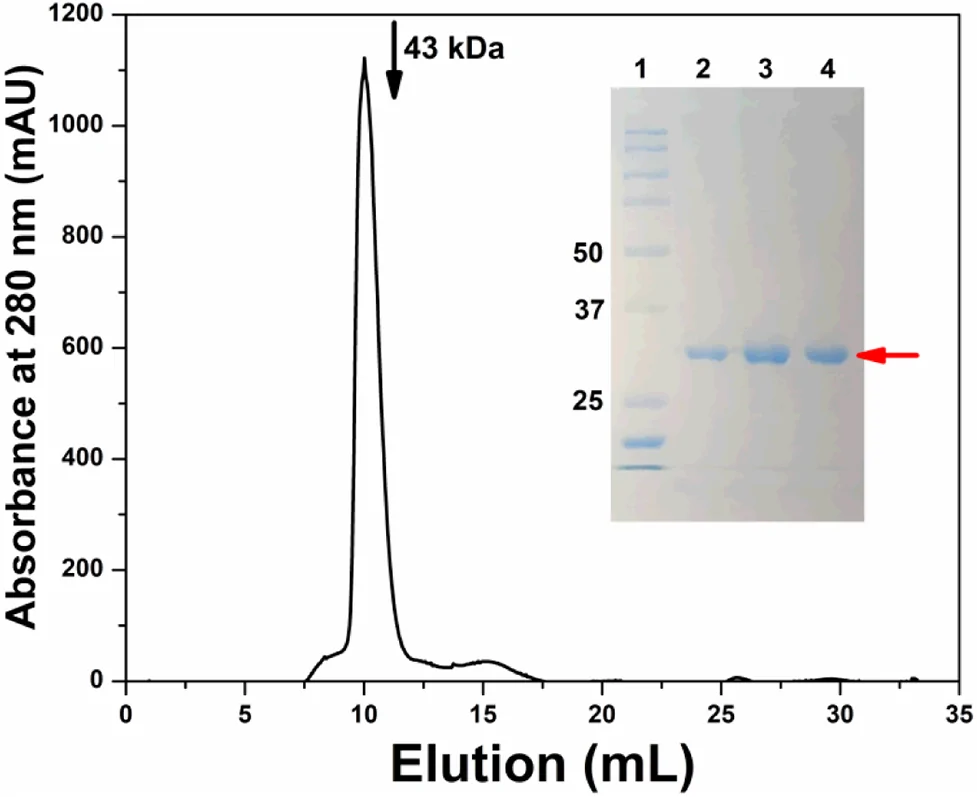
Size-exclusion chromatography (SEC) of recombinant TbEst. The black arrow indicates the elution volume corresponding to the molecular mass standard (43 kDa). Inset: Coomassie-stained SDS-PAGE of the following SEC purification. Lane 1: molecular-mass standards; lanes 2, 3, and 4: SEC fractions at 9, 10, and 11 mL. The red arrow indicates the TbEst monomer (∼29 kDa).

SDS-PAGE analysis under reducing conditions confirmed the high purity of recombinant TbEst, which appeared as a single protein band consistent with the predicted monomeric molecular mass of approximately 29 kDa (inset of Figure 2). Analytical size-exclusion chromatography under native conditions (Figure 2) revealed that TbEst eluted as a symmetrical peak earlier than the largest molecular mass standard (43 kDa), suggesting the formation of an oligomeric molecule in solution. Linear regression of the protein standards yielded an estimated apparent molecular mass of 62 kDa (Figure S2). In addition, the *R*_*h*_ determined for TbEst was 3.5 ± 0.3 nm, a value corresponding to an estimated molecular mass of 63 ± 12 kDa and consistent with a homodimer in solution. Furthermore, we used AlphaFold-Multimer to generate a predicted model of the TbEst homodimer (Figure S3). The model yielded ipTM = 0.94 and pTM = 0.95, indicating high confidence in both the overall fold and the interchain interface. The mean pLDDT was 97.93, with 97.2% of residues in the “very high confidence” category (Table S2). The predicted homodimer (Figure S3) adopts a head-to-head assembly with a buried surface area of ∼1509 Å^2^, stabilized by hydrophobic residues (Ile4, Phe17, Ile19, Ile43, Phe47, Pro50, Leu164, Leu230, Ile233) and hydrogen bonds or salt bridges (Arg15, His42, Thr46, Arg65, Asp67, Asp73, Ser74). Homodimeric esterases have been reported in other thermophilic organisms, such as the Dienelactone Hydrolase (HtDLH) esterase from the thermophilic bacterium *Hydrogenobacter thermophilus*.^17^ However, further analyses using high-resolution techniques are required to confirm the exact oligomeric state of TbEst in solution.

### 3.3 Effects of pH and Temperature on the Esterase Activity of Recombinant TbEst

Biochemical assays were conducted to assess TbEst’s esterase activity. The activity was first tested across different temperatures at pH 8.0 using pNPA as the substrate (Figure 3A). Under these conditions, TbEst showed maximum activity at 50 °C. Notably, TbEst maintained about 75% of its maximum activity at 70 °C. Next, the enzyme’s activity was measured at various pHs at its optimal temperature (Figure 3B), exhibiting maximum activity at pH 8.0 (Figure 3B). Overall, our results indicate that the optimal conditions for TbEst activity are 50 °C and pH 8.0.

**Figure 3:**
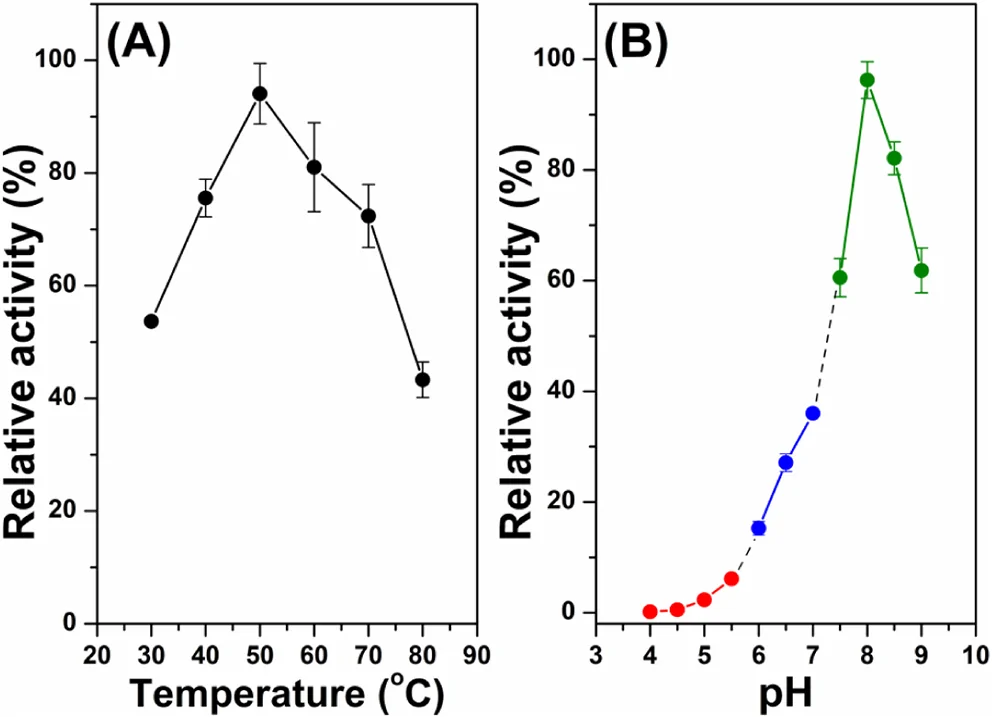
Esterase activity of recombinant TbEst using pNPA as the substrate. **A)** Temperature-dependent catalytic activity of recombinant TbEst at pH 8. **B)** pH-dependent catalytic activity of recombinant TbEst. Connecting lines are included only as a guide to the eye and do not represent fitted curves. Colors indicate buffer systems used: acetate buffer (red), sodium phosphate buffer (blue), and Tris-HCl buffer (green). Dashed lines indicating the boundaries between the different buffer systems.

The literature describes numerous thermostable, alkaline-active esterases with diverse origins.^21,49–52^ For instance, EstCS1 (esterase from compost metagenome library)^49^ and EprEst (esterase from deep-sea hydrothermal vent)^51^ both exhibit optimal activity at 50 °C and pH 8.0. Higher thermal stability is seen in the TLE esterase from *Thermomyces lanuginosus* ^21^ and the Ca-Est esterase from *Clostridium acetobutylicum*,^52^ both of which reach optimal activity at 60 °C under alkaline conditions. Meanwhile, the metagenomic recombinant lipolytic esterase EST5 operates at a slightly lower threshold of 45 °C and pH 7.5.^50^

The specific activities of TbEst against pNPA, pNPB, and pNPD were 25.8 ± 0.1 U/mg, 1.70 ± 0.07 U/mg, and 0.27 ± 0.04 U/mg, respectively. Moreover, the enzyme demonstrated the ability to degrade other aromatic esters (Figure S4), such as phenyl propionate (0.15 ± 0.05 U/mg), vinyl butyrate (0.28 ± 0.01 U/mg), cyclohexyl butyrate (0.36 ± 0.03 U/mg), and diethyl phthalate (0.18 ± 0.01 U/mg). In contrast, no significant hydrolysis was detected with pNPP as a substrate, although activity was observed on tripropionin (0.29 ± 0.01 U/mg), a short-chain triacylglycerol-like substrate (Figure S4). Additionally, detectable activity was seen on multiesterified substrates (Figure S4), including glyceryl tributyrate (0.36 ± 0.03 U/mg) and glyceryl triacetate (0.26 ± 0.01 U/mg). Collectively, these findings highlight the enzyme’s broad utility and biotechnological potential for catalyzing the biotransformation of ester-containing compounds. Compared to the *T. maritima* acetyl xylan esterase TM0077,^29^ which exhibits optimal activity at 70 °C and prefers short-chain substrates, TbEst shows broader substrate specificity (including aromatic esters) and a lower optimal temperature (50 °C). These contrasting catalytic profiles highlight evolutionary adaptation to distinct ecological niches among *Thermotogaceae* esterases. Finally, the kinetic parameters were obtained for pNPA hydrolysis (Figure S5). TbEst exhibited an estimated *V*_max_ and *K*_m_ of 1.1 μM s^–1^ and 0.9 mM, respectively. Additionally, *k*_cat_ = 207.5 s^–1^ and *k*_cat_/*K*_m_ = 230.6 mM^–1^ s^–1^. The *K*_m_ value estimated for TbEst was slightly lower than the 1.1 mM reported for the esterase EST5 using the same substrate.^50^

### 3.4 Effect of Temperature on the Structural Stability of Recombinant TbEst

CD spectroscopy was used to monitor temperature-dependent changes in the secondary structure of TbEst. At pH 8.0 and 20 °C, the CD spectrum of TbEst displayed pronounced negative ellipticities around 208 and 222 nm (Figure 4), indicative of a mixed α/β secondary structure and consistent with the molecular modeling predictions (Figure 1). As the temperature increased, a significant change in ellipticity at 222 nm was observed after 65 °C (inset of Figure 4), indicating that TbEst maintains its secondary structure stability between 20 and 65 °C, followed by a progressive loss of secondary structure and activity at higher temperatures. The *T*_m_ of 78 ± 1 °C, calculated as the midpoint of the thermal unfolding transition, underscores the excellent thermostability of TbEst. For comparison, the esterase EprEst^51^ exhibited a *T*_m_ of 61.3 °C, while the recombinant esterase Ca-Est exhibited a *T*_m_ of 67.7 °C.^52^

**Figure 4:**
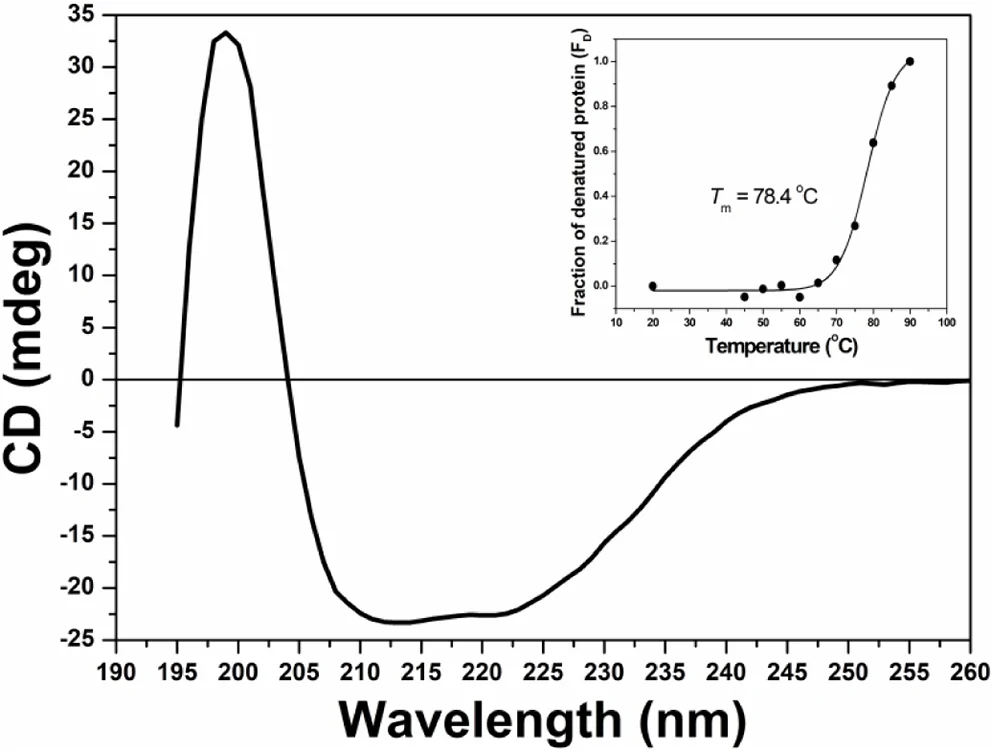
Circular dichroism (CD) spectrum of recombinant TbEst. Inset: thermal unfolding of TbEst monitored by circular dichroism (CD) spectroscopy at 220 nm.

To further investigate the mechanistic basis for the discrepancy between the optimal catalytic temperature (50 °C) and the *T*_m_ (78 °C), we analyzed the per-residue pLDDT scores from the AlphaFold model (Figure 1) as a proxy for structural flexibility. The catalytic triad residues (Ser113, Asp198, His225) exhibit a mean pLDDT of 98.43, which is higher than the overall mean of 97.03, indicating that the active-site core is exceptionally well-structured and rigid. To validate this observation with experimental data, we analyzed the crystallographic B-factors of two homologous *Thermotogaceae* esterases (PDB ID: 3DOH and 3M81). In both structures, the catalytic triad residues display mean B-factors lower than the overall protein average (29.56 vs 37.15 Å^2^ for 3DOH; 26.07 vs 27.59 Å^2^ for 3M81), confirming that the active-site core is also a rigid region in experimentally determined structures. TbEst contains regions with lower pLDDT values (minimum 78.25) and higher B-factors, suggesting the presence of flexible loops elsewhere in the structure. Based on these combined computational and experimental observations, we propose that thermal inactivation above 50 °C is not due to local unfolding of the catalytic triad, but rather to subtle conformational changes in distal flexible regions that propagate to the active site and disrupt catalysis. This hypothesis is consistent with the CD data (Figure 4), where global secondary structure loss is observed only above 65 °C.

### 3.5 Development and Characterization of a Bioconjugate Using a Photochemical Synthesis Method

TbEst was used to synthesize TbEst@Ag/AgCl-NPs through photochemical reduction employing a previously described methodology with minor modifications.^33–35^ When TbEst was incubated in Tris-HCl buffer (pH 8) containing Ag^+^ ions and illuminated with visible light, the initially clear solution (inset of Figure 5, vial 2) gradually changed to light brown within 60 min (inset of Figure 5, vial 3). This color change visually indicates the formation of TbEst@Ag/AgCl-NPs. In contrast, the control solution in the absence of TbEst exhibited a rapid color change upon illumination, forming a black precipitate within minutes (inset of Figure 5, vial 1). In our system, visible light played a crucial role in reducing Ag^+^ ions to metallic Ag^0^, while TbEst stabilized the bioconjugate, likely through a combination of electrostatic and steric interactions.^33^

**Figure 5:**
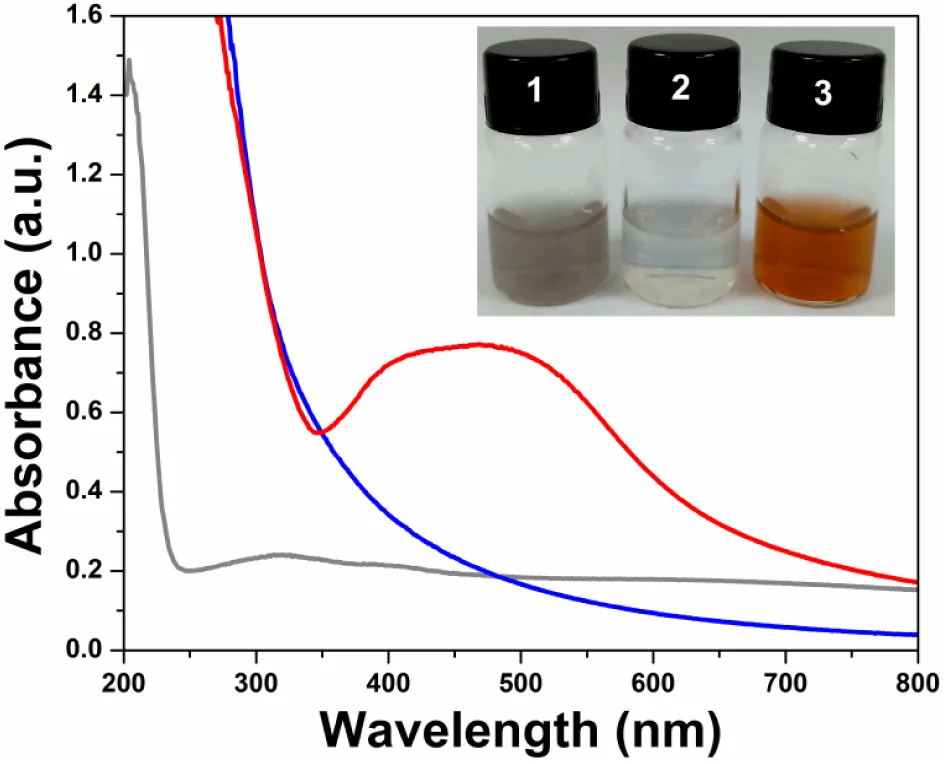
Absorption spectrum of TbEst@Ag/AgCl-NPs. Vial 1 (inset) and spectrum in gray - control: 1 mM Tris-HCl buffer, pH 8, containing 1 mM AgNO_3_ after 1 h illumination with visible light. Vial 2 (inset) and spectrum in blue: 1 mM Tris-HCl, pH 8, containing 1 mM AgNO_3_ and 0.1 mg/mL TbEst before illumination with visible light. Vial 3 (inset) and spectrum in red: 1 mM Tris-HCl buffer, pH 8, containing 1 mM AgNO_3_ and 0.1 mg/mL TbEst after 1 h illumination with visible light.

The synthesis of TbEst@Ag/AgCl-NPs was monitored spectrophotometrically (Figure 5). The observed strong absorption between 400 and 600 nm is attributed to the surface plasmon resonance (SPR) of the Ag NPs (Figure 5, spectrum in red).^53^ In the control solution, no characteristic absorption peak was observed between 400 and 500 nm (Figure 5, spectrum in gray), indicating that no NPs were produced. The TbEst@Ag/AgCl-NPs were purified by centrifugation and morphologically and chemically characterized prior to subsequent applications. The supernatant collected after immobilization and purification revealed no detectable protein when evaluated by the Bradford method, demonstrating efficient protein-nanoparticle association. Direct quantification of the pellet-bound TbEst was precluded by strong SPR interference at 280 nm, as well as silver-induced redox interference with standard colorimetric assays (such as the Bradford method). One month after purification, the color of the TbEst@Ag/AgCl-NPs suspension remained stable and displayed its characteristic SPR spectrum (Figure S6), suggesting long-term colloidal stability.

FTIR spectroscopy was employed to identify the functional groups within TbEst responsible for the reduction of silver ions and the subsequent stabilization of the synthesized Ag/AgCl-NPs. The spectrum of TbEst (Figure S7, black line) exhibited a characteristic absorption band near 1,650 cm^–1^, assigned to the Amide I (C=O stretching) vibration, which is consistent with the protein’s β-sheet secondary structure.^33,35^ The band observed around 1,550 cm^–1^ corresponds to the Amide II (C–N stretching and N–H bending) vibrations. Furthermore, the absorption band around 3,250 cm^–1^ is attributed to O–H stretching vibrations and confirms the presence of intermolecular hydrogen-bonding networks arising from the NH_2_ and OH groups within TbEst.^31,33^ The FTIR spectrum of the TbEst@Ag/AgCl-NPs (Figure S7, red line) displayed minimal deviations in the primary band pattern compared to isolated TbEst. The absorption band near 1,650 cm^–1^ is clearly present in both FTIR spectra. Furthermore, the presence of absorption bands between 1,030 cm^–1^ and 1,400 cm^–1^ in both FTIR spectra reinforces the presence of TbEst on the NP surface. These results confirm the successful association of TbEst onto the Ag/AgCl-NPs, where the protein acts as a robust capping and stabilizing agent.

XRD was used to analyze the crystal structure of the TbEst@Ag/AgCl-NPs. The XRD pattern (Figure 6) exhibited well-defined diffraction peaks around 2θ = 27.8°, 32.2°, 46.2°, 54.8°, 57.5°, 67.5°, and 74.5°, corresponding to the (111), (200), (220), (311), (222), (400), and (311) crystallographic planes of AgCl. These results confirm the formation of a crystalline AgCl phase, in agreement with values reported in the literature.^33–35^

**Figure 6:**
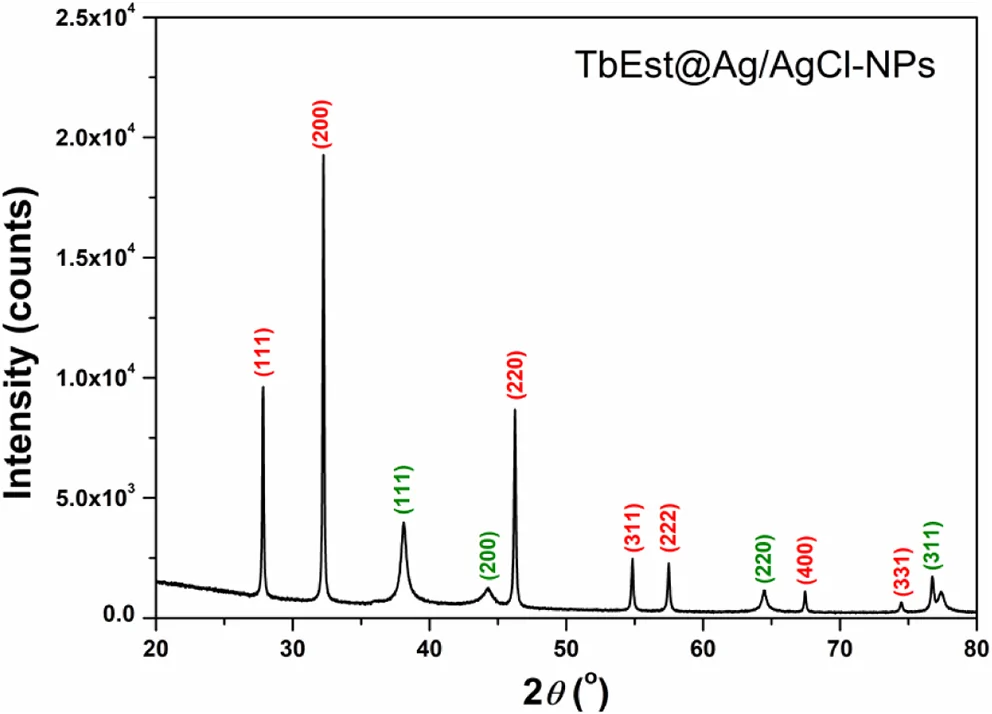
X-ray diffraction (XRD) pattern of TbEst@Ag/AgCl-NPs. The Miller indices are shown in red and green for AgCl and Ag^0^, respectively.

Additionally, well-defined diffraction peaks at 2θ = 38.1°, 44.3°, 64.5°, and 76.8°, corresponding to the (111), (200), (220), and (331) reflections, are attributed to metallic silver (Ag^0^). Taken together, these findings confirm the presence of a cubic AgCl crystalline phase coexisting with a smaller fraction of Ag-NPs, the latter responsible for the observed SPR effect.^33,35^

The TbEst@Ag/AgCl-NPs exhibited an *R*_h_ of 73 ± 3 nm in solution, with a corresponding zeta potential of −33 ± 2 mV, a highly negative value that confirms the robust colloidal stability of the bioconjugate.^37,43^ Also, the size and shape of the TbEst@Ag/AgCl-NPs were analyzed by TEM. The acquired micrograph displayed agglomeration of NPs as well as dispersed NPs (Figure 7A).

**Figure 7:**
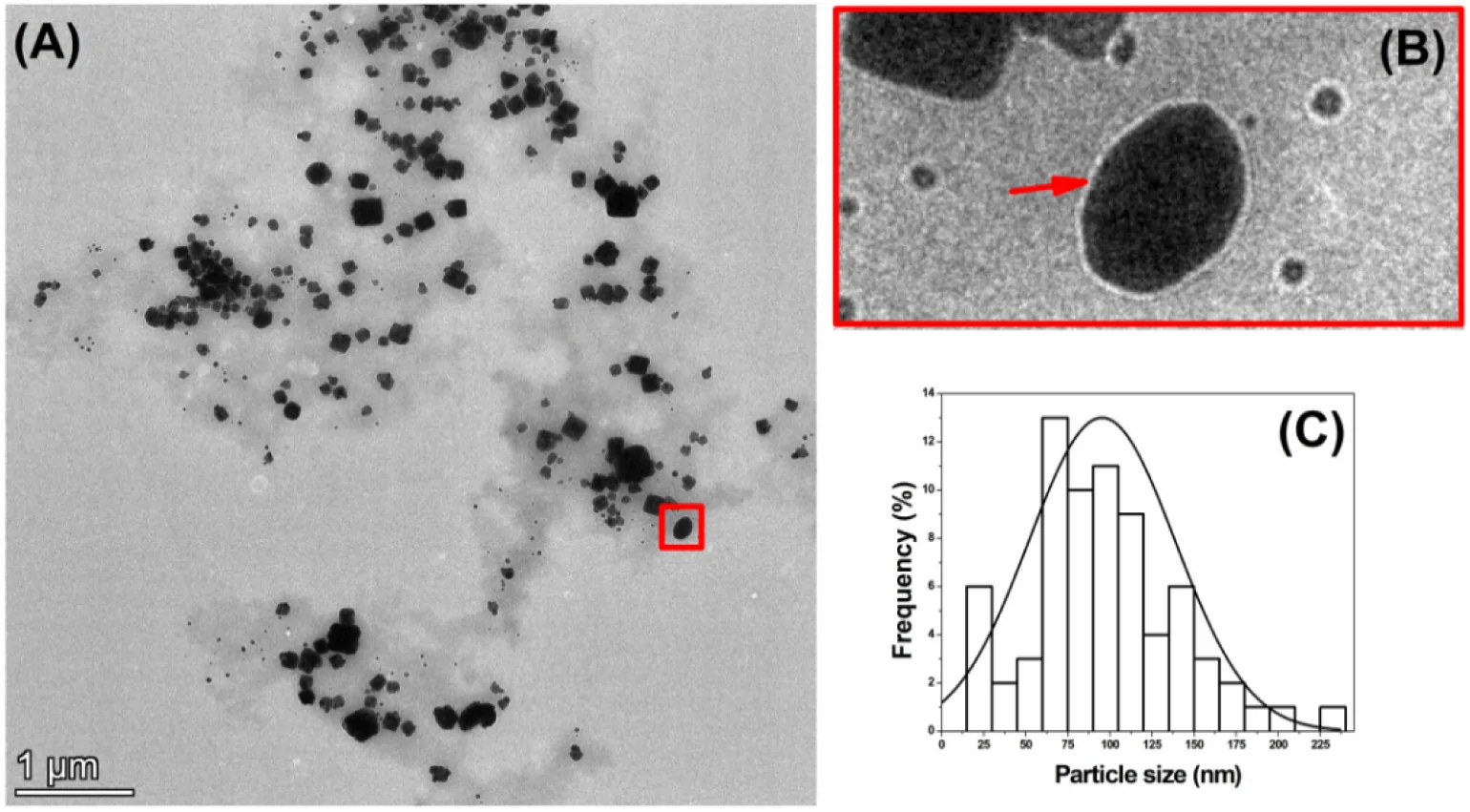
Transmission electron microscopy (TEM) image of TbEst@Ag/AgCl-NPs. **A)** A representative transmission electron micrograph showing purified TbEst@Ag/AgCl-NPs. **B)** Magnified image providing a detailed view of the region delimited by the red box in panel A. The red arrow indicates the TbEst corona over the NPs. **C)** Particle size distribution histogram of TbEst@Ag/AgCl-NPs. The total number of NPs counted in each case was 72.

Micrographs also reveal two predominant morphologies for the TbEst@Ag/AgCl-NPs: spherical and cubic (Figure 7A and Figure S8). Furthermore, Figure 7B provides a detailed view of the region delimited by the red box in panel A. The dark, high-contrast core corresponds to the inorganic fraction due to its higher electron density, while the surrounding lighter, semitransparent halo (indicated by the red arrow) is attributed to the organic TbEst coating (Figure 7B). The particle size distribution histogram (Figure 7C) indicates that the TbEst@Ag/AgCl-NPs range in size from 20 to 200 nm, with a mean diameter of 100 nm, with a polydispersity that directly corroborates the broad absorption spectrum observed in Figure 5. These anisotropic NPs possess unique physicochemical properties that hold promise for applications in optical sensing, imaging, photonics, electronics, catalysis, and electronic devices.^33^ Finally, EDS analysis confirmed the presence of Ag and Cl from the AgCl semiconductor, as well as C and O from the immobilized TbEst (Figure S9).

### 3.6 Enhanced Thermostability of TbEst Following Nanoscale Immobilization

While free enzymes offer potential biotechnological and environmental applications, they often face challenges such as unfolding, low thermal stability, and limited reusability. An adequate platform utilized for immobilization can provide physical and chemical properties to overcome these limitations through its increased activity, stability, and potential reusability.^54^ To evaluate whether catalytic activity was retained in the TbEst@Ag/AgCl-NPs, esterase activity was measured using pNPA as the substrate (Figure 8). Following photochemical synthesis, the TbEst@Ag/AgCl-NPs were collected by centrifugation, washed, and resuspended to their initial volume. The TbEst@Ag/AgCl-NPs recovered approximately 67% of the activity compared to the free enzyme. This decrease in activity likely stems from surface-induced structural adjustments and orientation constraints required for TbEst to coordinate Ag^+^ ions and cap the NPs, which partially distort or obstruct active sites in a fraction of the immobilized enzyme population.

**Figure 8:**
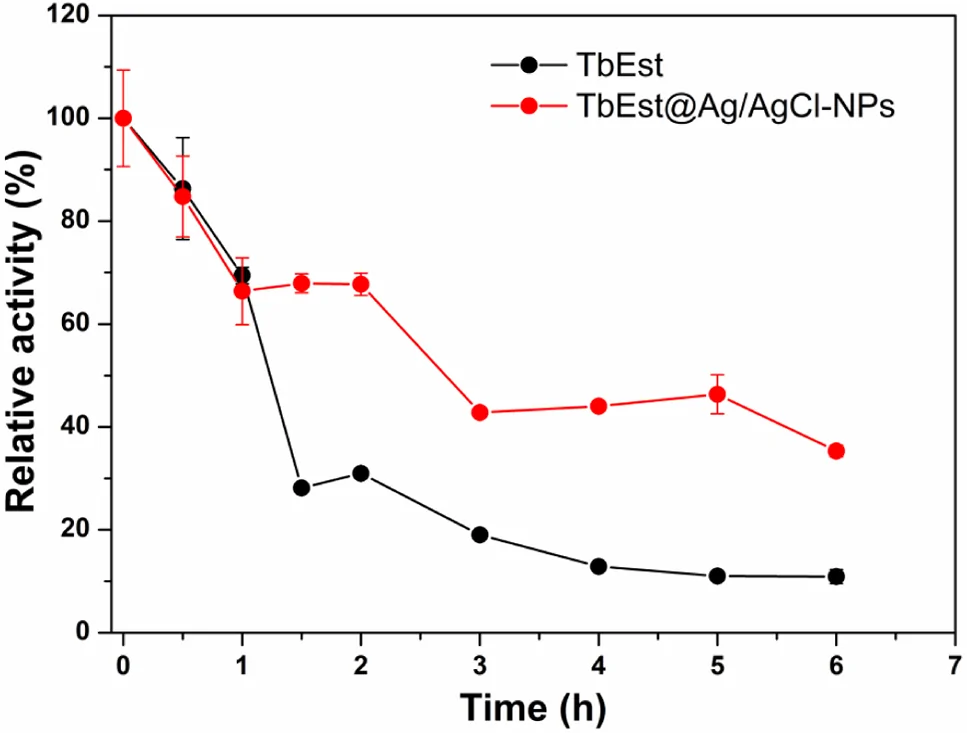
Thermostability of free TbEst and TbEst@Ag/AgCl-NPs. Black line (control): free TbEst was incubated at 60 °C in 20 mM Tris-HCl buffer, pH 8, for 6 h. Red line: TbEst@Ag/AgCl-NPs bioconjugate was incubated at 60 °C in 20 mM Tris-HCl, pH 8, for 6 h. Connecting lines are included only as a guide to the eye and do not represent fitted curves.

Figure 3A shows that TbEst reached its optimal activity at 50 °C, retaining approximately 81% of its relative activity at 60 °C. To further evaluate the comparative thermal stability of free TbEst and TbEst@Ag/AgCl-NPs, their residual activities were monitored over time at 60 °C (Figure 8).

During the first hour of incubation, both preparations exhibited a similar inactivation profile, retaining approximately 70% of their initial activity. However, a significant divergence was observed thereafter. While the free TbEst exhibited a sharp decrease in its residual activity, TbEst@Ag/AgCl-NPs demonstrated a superior stability plateau. By the end of the 6-h period, the free TbEst retained only approximately 10% of its activity, whereas the TbEst@Ag/AgCl-NPs bioconjugate maintained roughly 40%, representing a nearly 4-fold increase in residual activity compared to the free form (Figure 8).

These results corroborate previous reports indicating that trypsin immobilization onto biogenic Ag-NPs effectively preserves catalytic activity.^55^ Consistent with our findings, a similar study on the immobilization of *Trichoderma harzianum* α-amylase onto Ag-NPs reported a significant enhancement in thermal stability. When evaluated across a temperature gradient (30 to 80 °C), the immobilized α-amylase exhibited superior activity performance relative to the free enzyme.^56^ Additionally, the immobilized cellulase from *Aspergillus niger* retained approximately 80% of its initial activity when compared to the free enzyme after a 1 h.^57^

The bioconjugation of enzymes, such as TbEst, with Ag/AgCl-NPs effectively stabilizes their structural integrity. The enhancement in the thermostability of nanoimmobilized TbEst can be explained by a coordinated mechanism of electrostatic alignment and rigid anchoring.^58,59^ After photochemical synthesis, TbEst molecules are distributed on the NP surface, ensuring optimal structural orientation. By anchoring to a rigid inorganic scaffold, the enzymes are spatially isolated and sterically hindered, restricting the molecular mobility required for unfolding and increasing the activation energy barrier of denaturation. This nanoscale immobilization strategy not only preserves catalytic activity and increases thermostability but also significantly broadens the industrial and biotechnological utility of the enzyme.

The reusability of TbEst@Ag/AgCl-NPs was evaluated across consecutive catalytic cycles, showing that the catalyst retained 70% of its initial activity after three cycles (Table S3). However, from the fourth cycle onward, reusability was limited by physical stability issues, with the formation of irreversible macroscopic aggregates hindering dispersion and resulting in an abrupt loss of activity. This sharp decline in activity after the fourth cycle stems primarily from mechanical compaction during centrifugal recovery rather than intrinsic colloidal instability. The TbEst@Ag/AgCl-NPs provide excellent operational stability, facilitate easy recovery and reuse of the catalyst, and prevent premature denaturation. Furthermore, this hybrid system merges the specific esterase activity of TbEst with the unique physicochemical traits of silver-based NPs, opening doors for specialized applications in bioremediation and biocatalysis, where free enzymes fail to persist.

## 4 Conclusion

This study describes the comprehensive characterization and nanoscale immobilization of TbEst, a novel thermophilic esterase from the family *Thermotogaceae*. Structural modeling confirmed that TbEst adopts a classic α/β-hydrolase fold featuring a conserved Ser-His-Asp catalytic triad. TbEst exhibited a high *T*_m_ value, underscoring its robust thermophilic nature. Biochemical assays identified optimal catalytic activity for TbEst at 50 °C and pH 8.0, along with activity against various aliphatic and aromatic ester substrates, supporting the enzyme’s versatility in biotechnological applications. The TbEst@Ag/AgCl-NPs bioconjugate displayed spherical and cubic morphologies with an average diameter of 100 nm, and a high negative zeta potential, ensuring excellent colloidal stability. Notably, nanoscale immobilization of TbEst onto Ag/AgCl-NPs significantly enhanced the enzyme thermostability, enabling longer retention of activity at 60 °C compared to the free enzyme. This enhanced thermostability, combined with the unique optical and physical properties of Ag/AgCl-NPs, highlights the potential of this bioconjugate for various applications. The data presented herein provide a useful basis for exploring the multifaceted applications of TbEst@Ag/AgCl-NPs across biocatalysis, bioremediation, and nanotechnology.

## Supplementary Material



## Notes

Complete amino acid sequence of TbEst (Table S1); validation metrics for the TbEst homodimer model (Table S2); reuse cycles of TbEst@Ag/AgCl-NPs (Table S3); structural superimposition of TbEst with known esterases (Figure S1); Kav versus log­(MM) plot (Figure S2); AlphaFold-Multimer predicted model of the TbEst homodimer (Figure S3); relative enzymatic activity of TbEst across different substrates (Figure S4); Michaelis–Menten plot (Figure S5); absorption spectrum of TbEst@Ag/AgCl-NPs measured 1 day and 1 month after synthesis (Figure S6); spectra of Fourier-transform infrared spectroscopy (FTIR): gray line—spectrum of free TbEst and black line—spectrum of TbEst@Ag/AgCl-NPs (Figure S7); transmission electron microscopy (TEM) of TbEst@Ag/AgCl-NPs (Figure S8); and energy-dispersive spectroscopy (EDS) spectrum reveals the components (Ag, Cl, C, and O) of TbEst@Ag/AgCl-NPs (Figure S9) (PDF)
