# Supplementary material for: Characterization and Nanoparticle Immobilization of a Thermotogaceae Esterase Active on Aromatic and Aliphatic Esters
Source: ACS Omega. 2026 Oct 7;11(41):62944–54. doi: 10.1021/acsomega.6c05063 (PMC13689099; doi:10.1021/acsomega.6c05063)
Supplement: Supplementary file 1 [file ao6c05063_si_001.pdf]

Supplementary Material

Characterization and nanoparticle immobilization of a *Thermotogaceae* esterase active on aromatic and aliphatic esters

Pietra Bruna Barbosa Bento[a], Dnane Vieira Almeida[a], Iara Cianglini[b], Ellen K.B. Roman[b], João Renato Carvalho Muniz[c], Roberta Albino dos Reis[a], Fabio Marcio Squina[b], Wanius Garcia[a,*]

[a] Centro de Ciências Naturais e Humanas, Universidade Federal do ABC (UFABC), CEP 09210-170, Santo André, SP, Brazil.

[b] Laboratory of Molecular Sciences, University of Sorocaba (UNISO), CEP 18060-501, Sorocaba, SP, Brazil.

[c] São Carlos Institute of Physics (IFSC), University of São Paulo (USP), CEP 13563-590, São Carlos, SP, Brazil.

[*] Corresponding author: wanius.garcia@ufabc.edu.br

Table S1. Complete amino acid sequence of TbEst. **02**

Table S2. Validation metrics for the TbEst homodimer model. **03**

Table S3. Reuse cycles of TbEst@Ag/AgCl-NPs. **04**

Figure S1. Structural superimposition of TbEst with known esterases. **05**

Figure S2. Kav versus log(MM) plot. **06**

Figure S3. AlphaFold-Multimer predicted model of the TbEst homodimer. **07**

Figure S4. Relative enzymatic activity of TbEst across different substrates. **08**

Figure S5. Michaelis–Menten plot. **09**

Figure S6. Absorption spectrum of TbEst@Ag/AgCl-NPs measured 1 day and 1 month after the synthesis. **10**

Figure S7. Spectra of Fourier-transform infrared spectroscopy (FTIR). **11**

Figure S8. Transmission electron microcopy (TEM) of TbEst@Ag/AgCl-NPs. **12**

Figure S9. Energy dispersive spectroscopy (EDS). **13**

Table S1

>Thermotogaceae bacterium 3_Gene A0A7X7W8F2
MSRIESFTTGLDGNRIFGIFELPEGGDSFPVVVMLHGFTGEHIVSTFKYPRLSRKLVKRGIATVRFDF
RGSGDSEGEFCDMSPLTELRDALEIVSLVREKSWFNGKLALIGY**S**MGGMVASLLAGREREFDAVLL
WSPVIMNGEFFSREEYSFSRDEKYKDILGLKLGPGFQRDGLSVDASAELENYHGPLMIIHGSE**D**ES
VPYEPVMRYARERGIEFHTVKGAN**H**KYQRLDWIEELFEVSERFLCDQLL

Table S1. Complete amino acid sequence of TbEst. In UniProt database the TbEst corresponds to the gene locus GX457_08790 and is documented under entries UPI000B065873 and A0A7X7W8F2. The amino acid residues that form the catalytic triad (Ser113, His225, and Asp198) are highlighted in bold. The conserved Gly111-Tyr112-**Ser113**-Met114-Gly115-Gly116 motif this is underlined.

Table S2

Metric	Value	Interpretation
ipTM	0.94	High confidence in inter-chain interface
pTM	0.95	High confidence in overall fold
Mean pLDDT	97.93	Very high average per-residue confidence
Min pLDDT	76.36	Good confidence (flexible loop regions)
Max pLDDT	98.99	Extremely high confidence (core)
Residues with pLDDT > 90	97.2%	Very high confidence
Residues with pLDDT 70–90	2.8%	Good confidence
Residues with pLDDT 50–70	0.0%	Low confidence (none)
Residues with pLDDT < 50	0.0%	Very low confidence (none)

Table S2. Validation metrics for the TbEst homodimer model predicted by AlphaFold-Multimer.

Table S3

Catalytic cycles	Retained activity (%)
-	100.0 ± 6.6
1	91.7 ± 4.9
2	83.4 ± 3.9
3	71.3 ± 3.1
4	39.9 ± 4.3

Table S3. Reuse cycles of TbEst@Ag/AgCl-NPs.

Figure S1

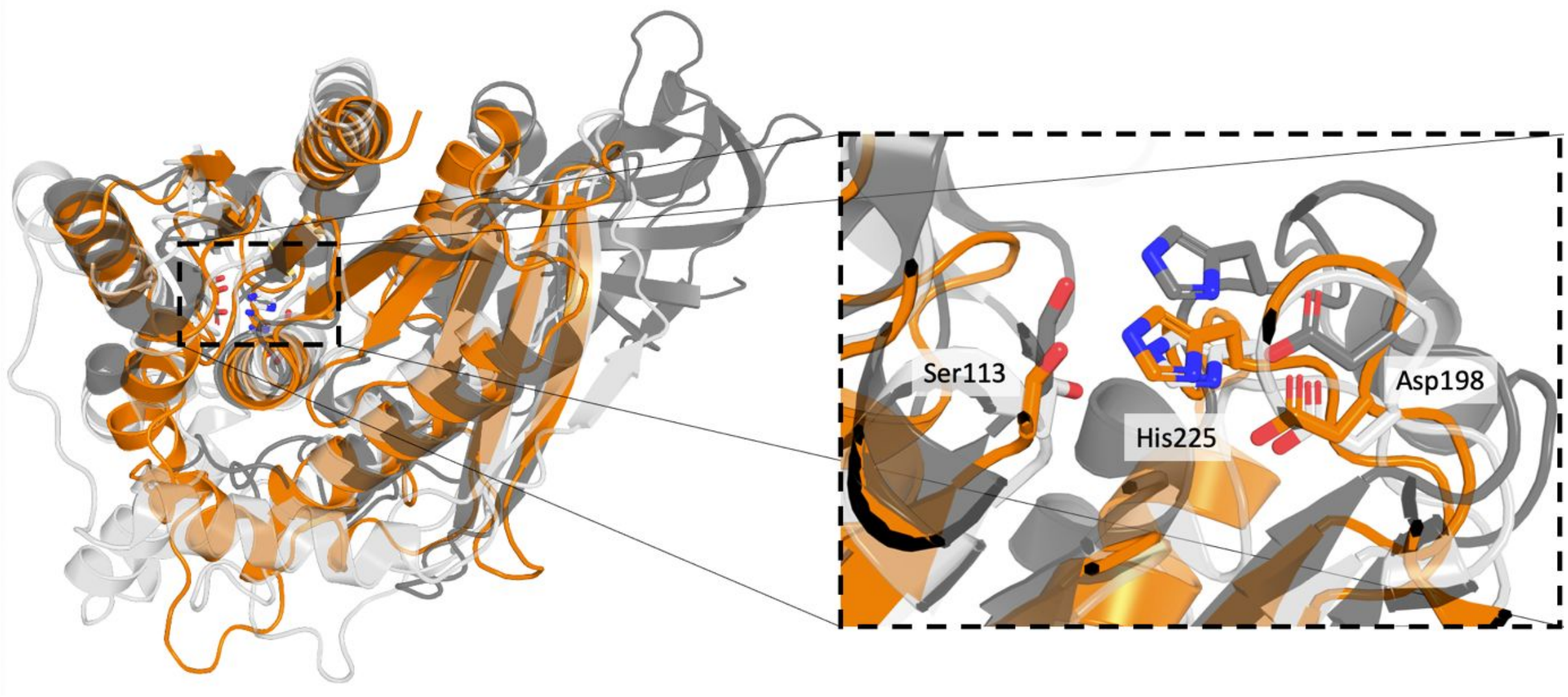

Figure S1. Structural superposition of the TbEst AlphaFold model with two experimentally determined Thermotogaceae esterases**. Left panel**: overall superposition of the TbEst monomer (orange) with EstA from *Thermotoga maritima* (PDB ID: 3DOH, dark gray) and acetyl xylan esterase TM0077 (PDB ID: 3M81, light gray). The superpositions were performed using the super algorithm in PyMOL, which aligns structures based on structural similarity of the conserved α/β-hydrolase core. RMSD values over the core region were 2.9 Å (vs. 3DOH) and 2.5 Å (vs. 3M81), confirming the high structural conservation of the central fold. The catalytic triad residues (Ser113, Asp198, His225) are highlighted by a dashed rectangle. The equivalent residues in the superimposed structures are Ser286, Asp334, His374 (PDB ID: 3DOH) and Ser188, Asp274, His303 (PDB ID: 3M81). **Right panel:** close-up view of the region delineated by the dashed rectangle, showing the superimposed catalytic triad residues. The side chains of Ser113, Asp198, and His225 are shown as sticks. The conserved spatial arrangement of the Ser-His-Asp triad, characteristic of the α/β-hydrolase superfamily, is clearly maintained in the TbEst model. Single chains were used for each superposition to enable a direct comparison of the monomeric α/β-hydrolase core.

Figure S2

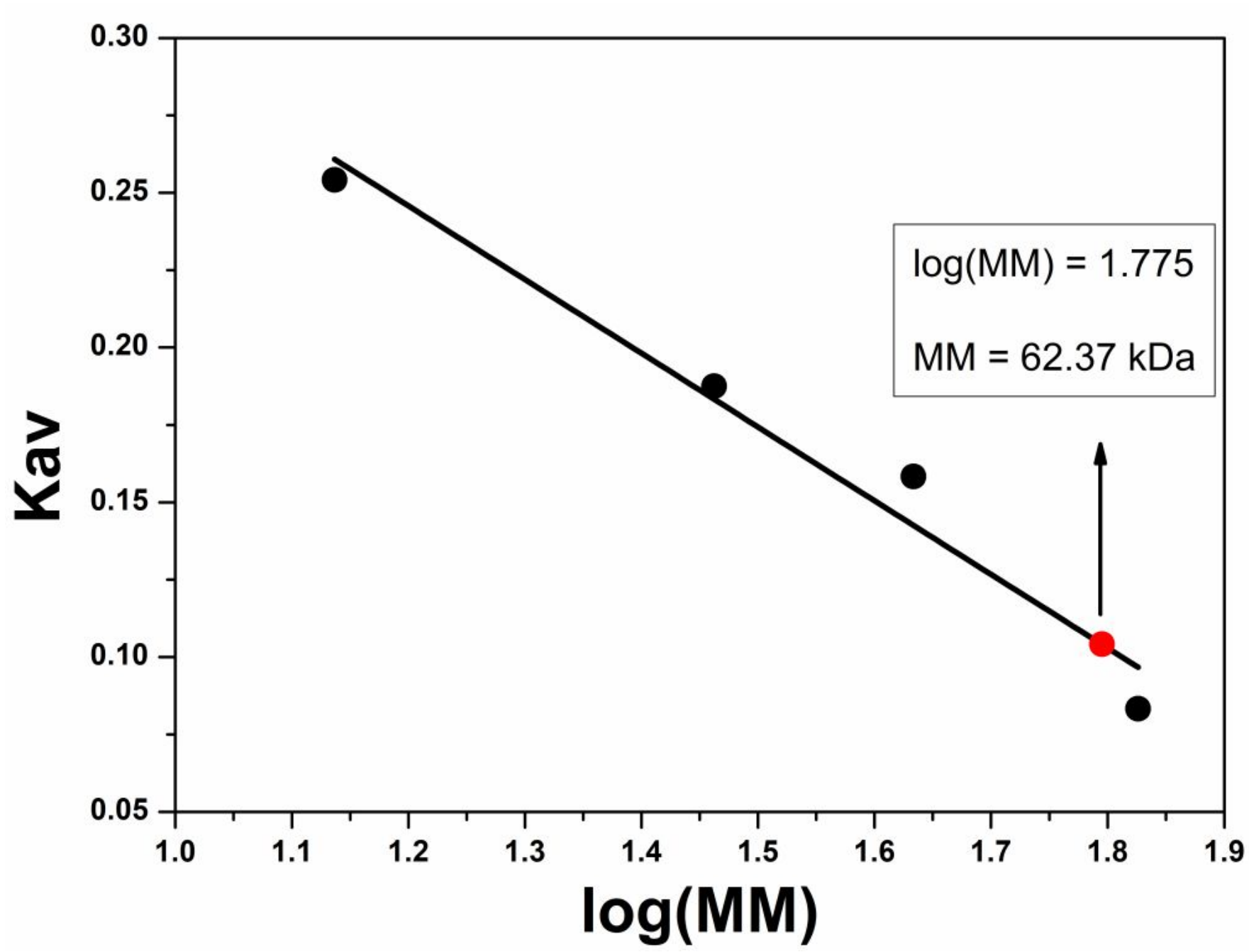

Figure S2. Size-exclusion chromatography (SEC) of purified TbEst on Superdex-75. The elution volumes of standard proteins were used to calculate the Kav values, where Kav = (elution volume – column void volume)/(total column volume – column void volume). The standard proteins of known molecular mass (MM) were BSA - bovine serum albumin (67 kDa), ovoalbumin (43 kDa), carbonic anhydrase (29 kDa), and ribonuclease A (13.7 kDa).

Figure S3

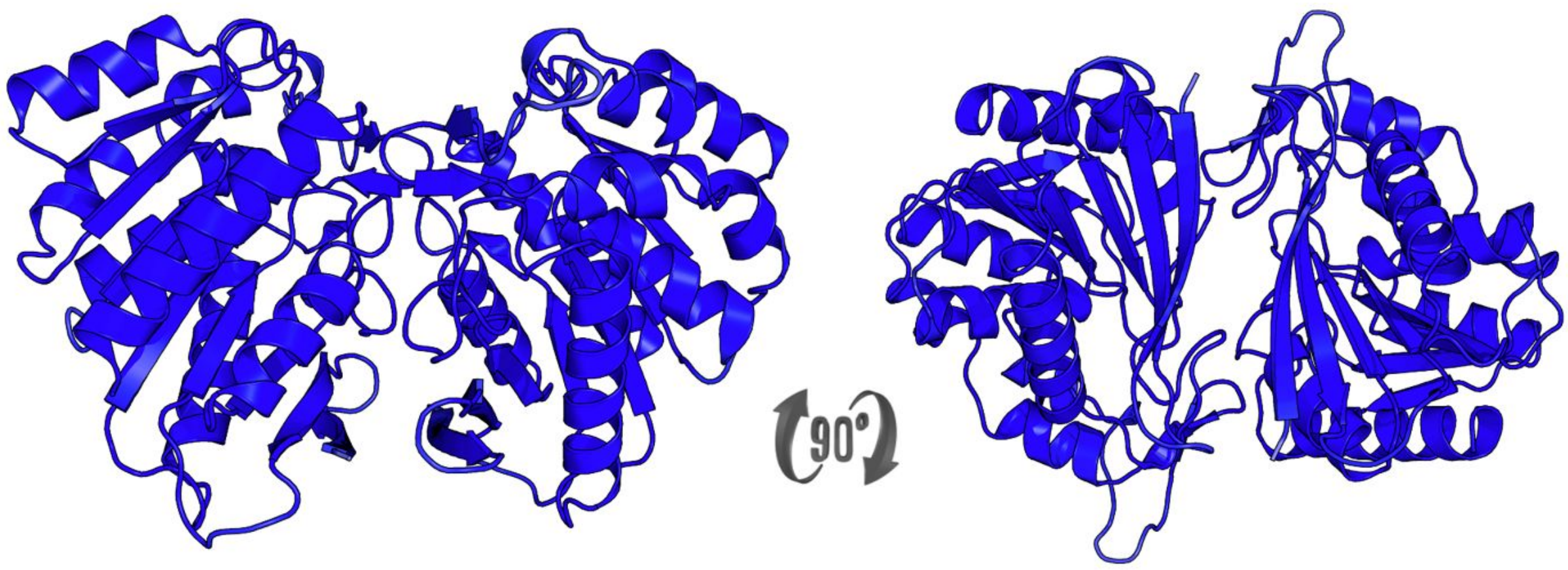

Figure S3. AlphaFold-Multimer predicted model of the TbEst homodimer, colored by pLDDT (predicted Local Distance Difference Test). The model is color-coded from blue (highest confidence, pLDDT > 90) to red (lowest confidence, pLDDT < 50). The high average pLDDT of 97.93, with 97.2% of residues in the 'very high confidence' category (pLDDT > 90), indicates that the vast majority of the structure is predicted with high confidence. The model yielded ipTM = 0.94 and pTM = 0.95, indicating high confidence in both the inter-chain interface and the overall fold.

Figure S4

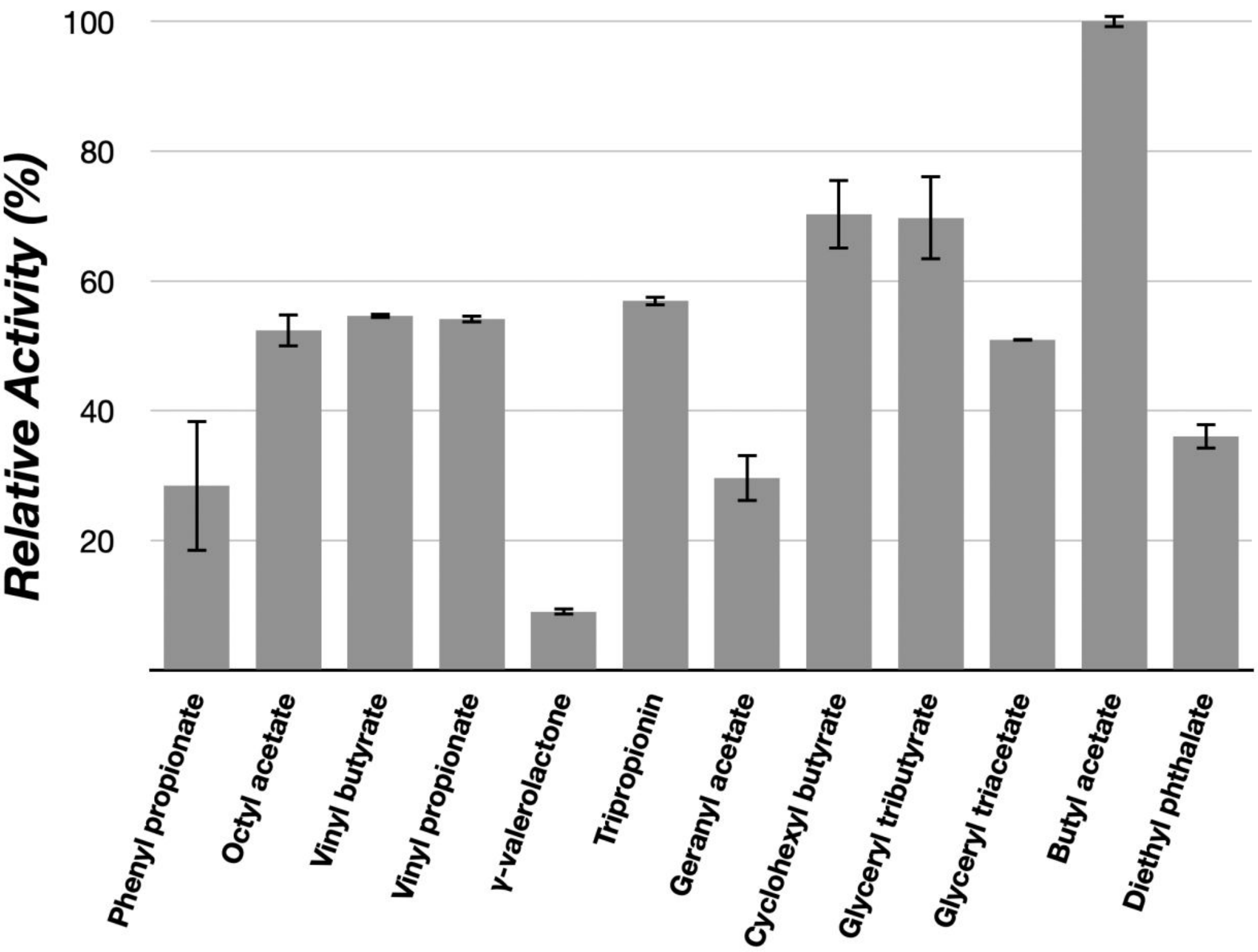

Figure S4. Relative enzymatic activity of TbEst across different substrates.

Figure S5

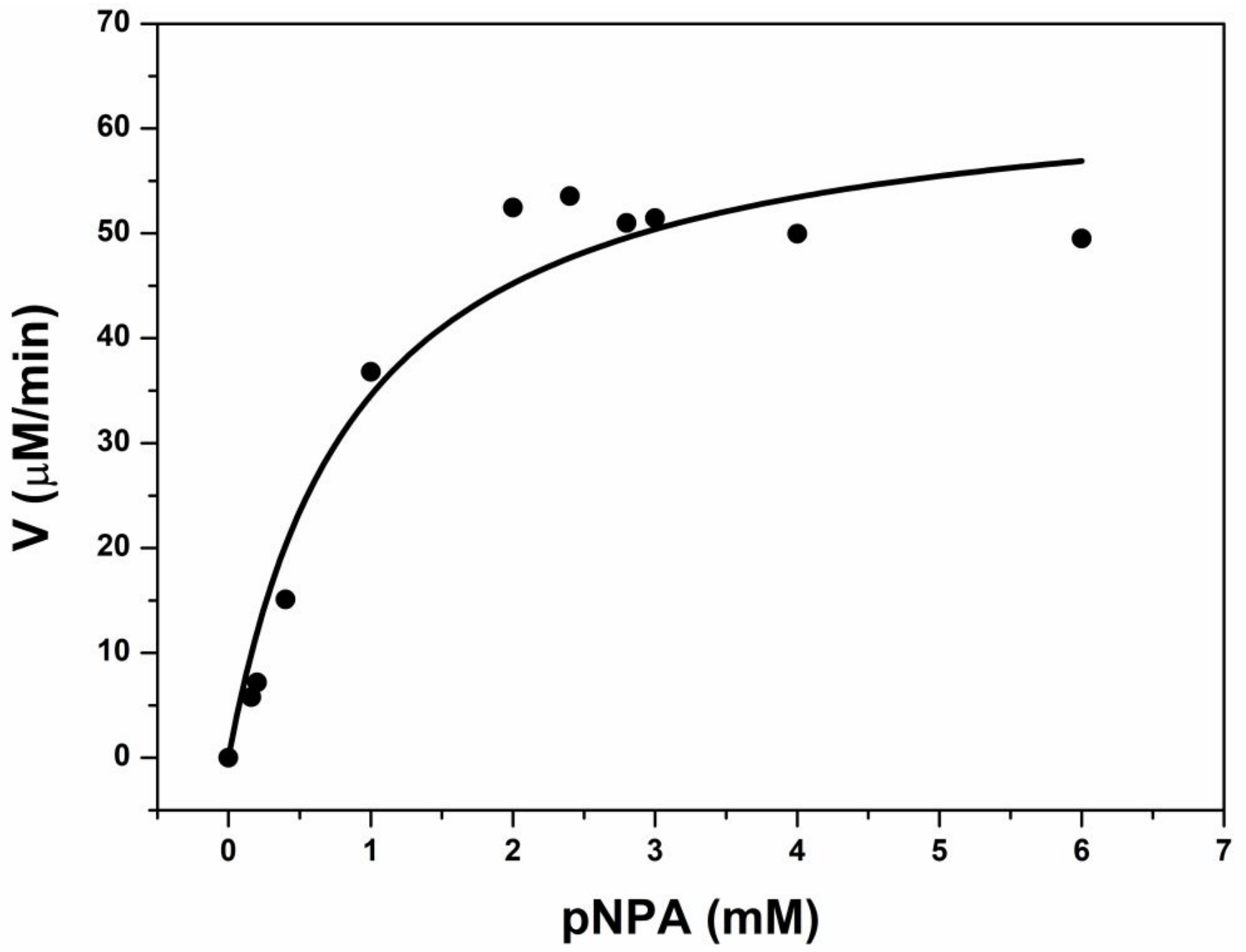

Figure S5. Michaelis–Menten plot of the initial reaction velocity as a function of the substrate concentration. The parameters K_m and V_{max} were determined by non-linear fitting of a rectangular hyperbole to the experimental points.

Figure S6

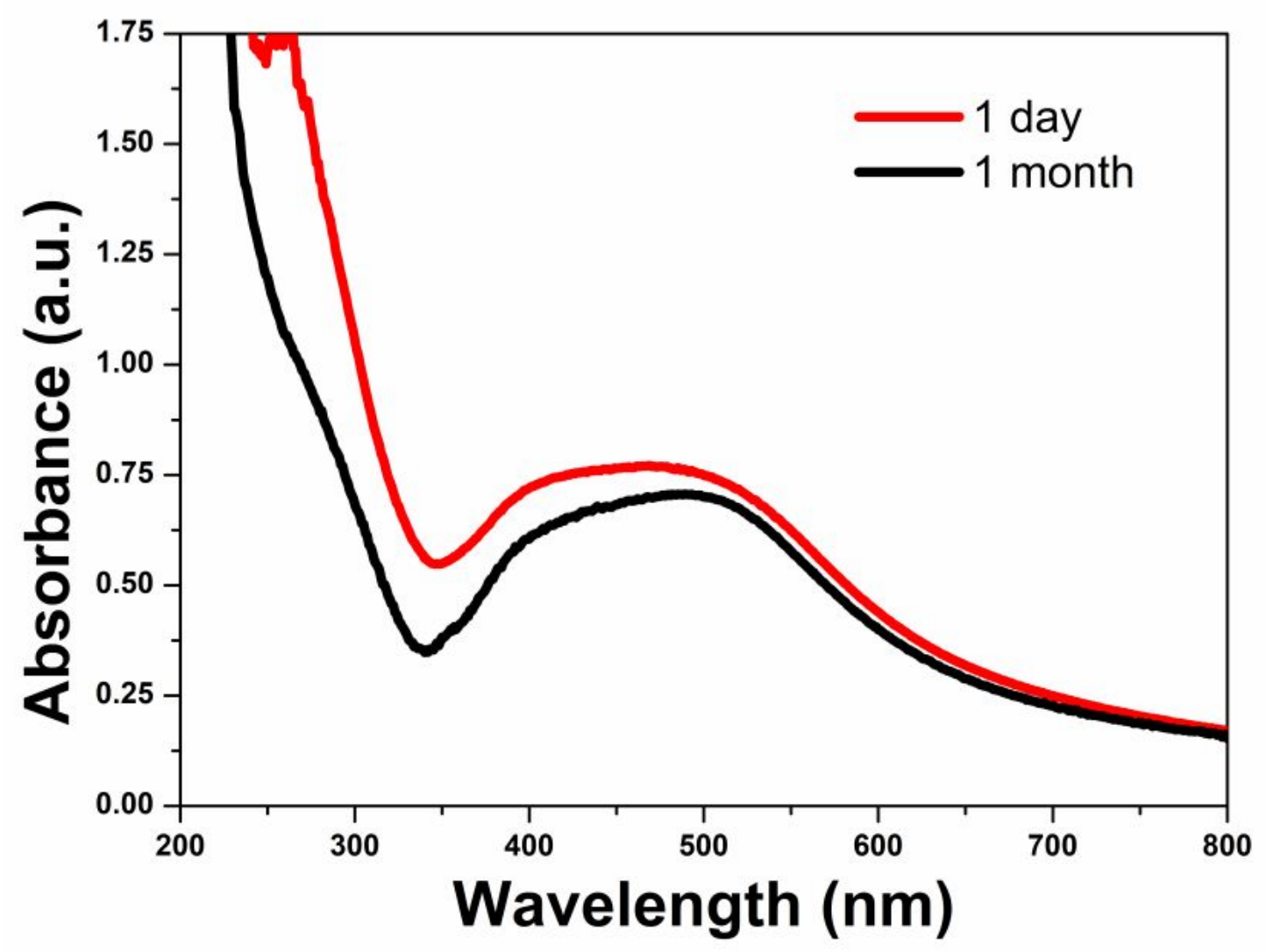

Figure S6. Absorption spectrum of TbEst@Ag/AgCl-NPs measured 1 day (red line) and 1 month (black line) after the synthesis.

Figure S7

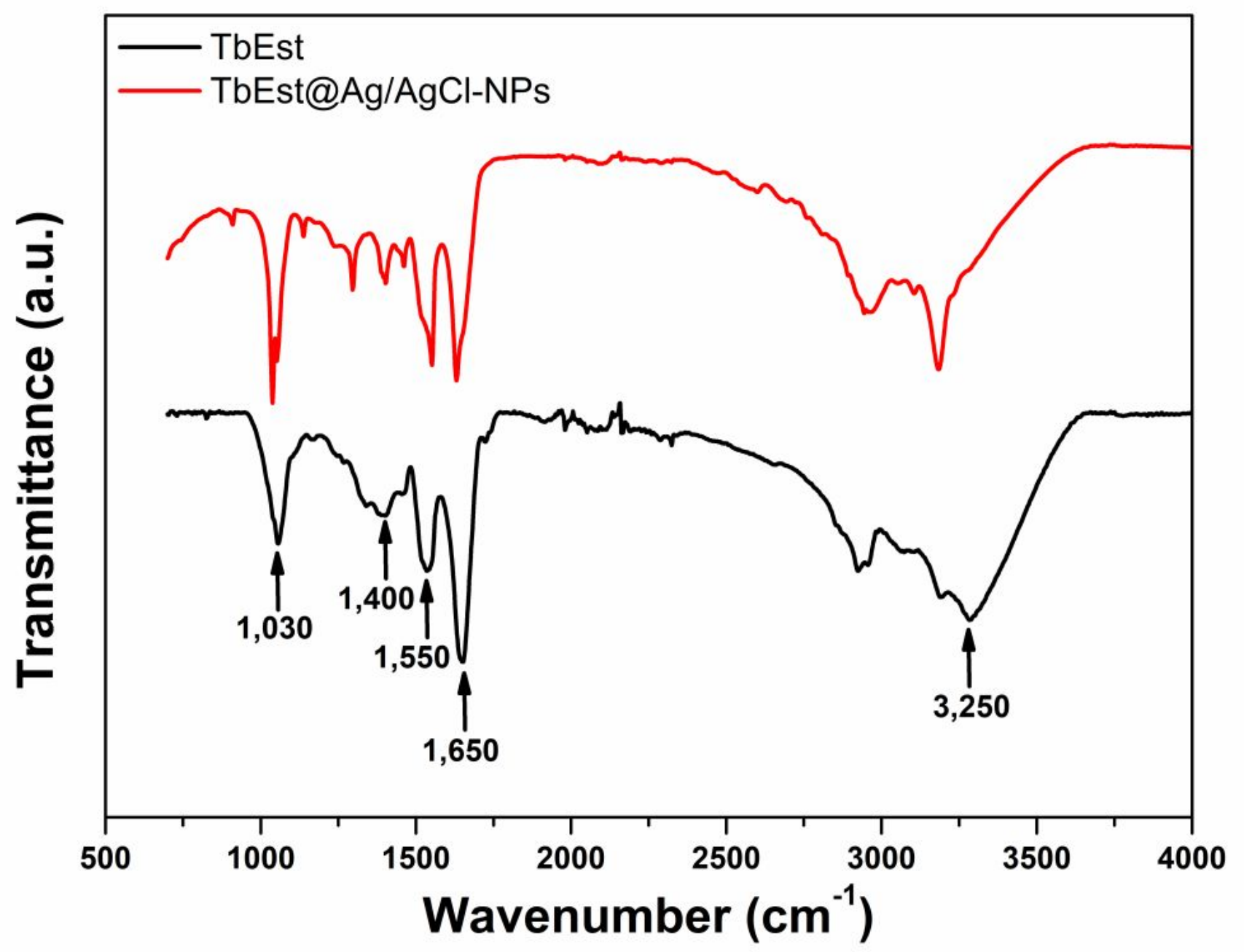

Figure S7. Spectra of Fourier-transform infrared spectroscopy (FTIR) – spectrum in black: free TbEst; spectrum in red: TbEst@Ag/AgCl-NPs.

Figure S8

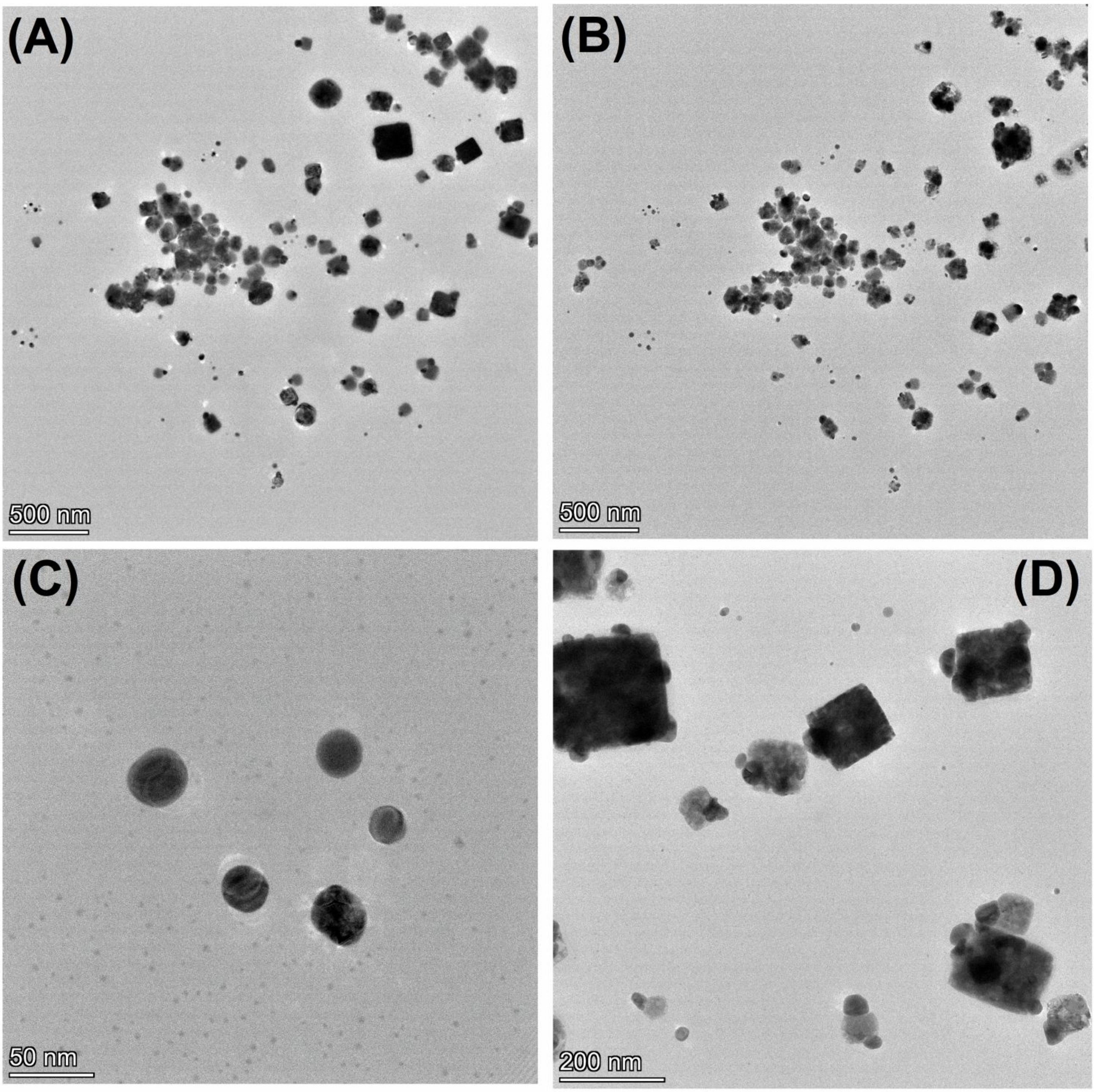

Figure S8. Transmission electron microcopy (TEM) of TbEst@Ag/AgCl-NPs. (**A)** and **(B)** Representative transmission electron micrographs showing purified TbEst@Ag/AgCl-NPs. **(C)** TbEst@Ag/AgCl-NPs with spherical shape. (**D)** TbEst@Ag/AgCl-NPs with cubic shape.

Figure S9

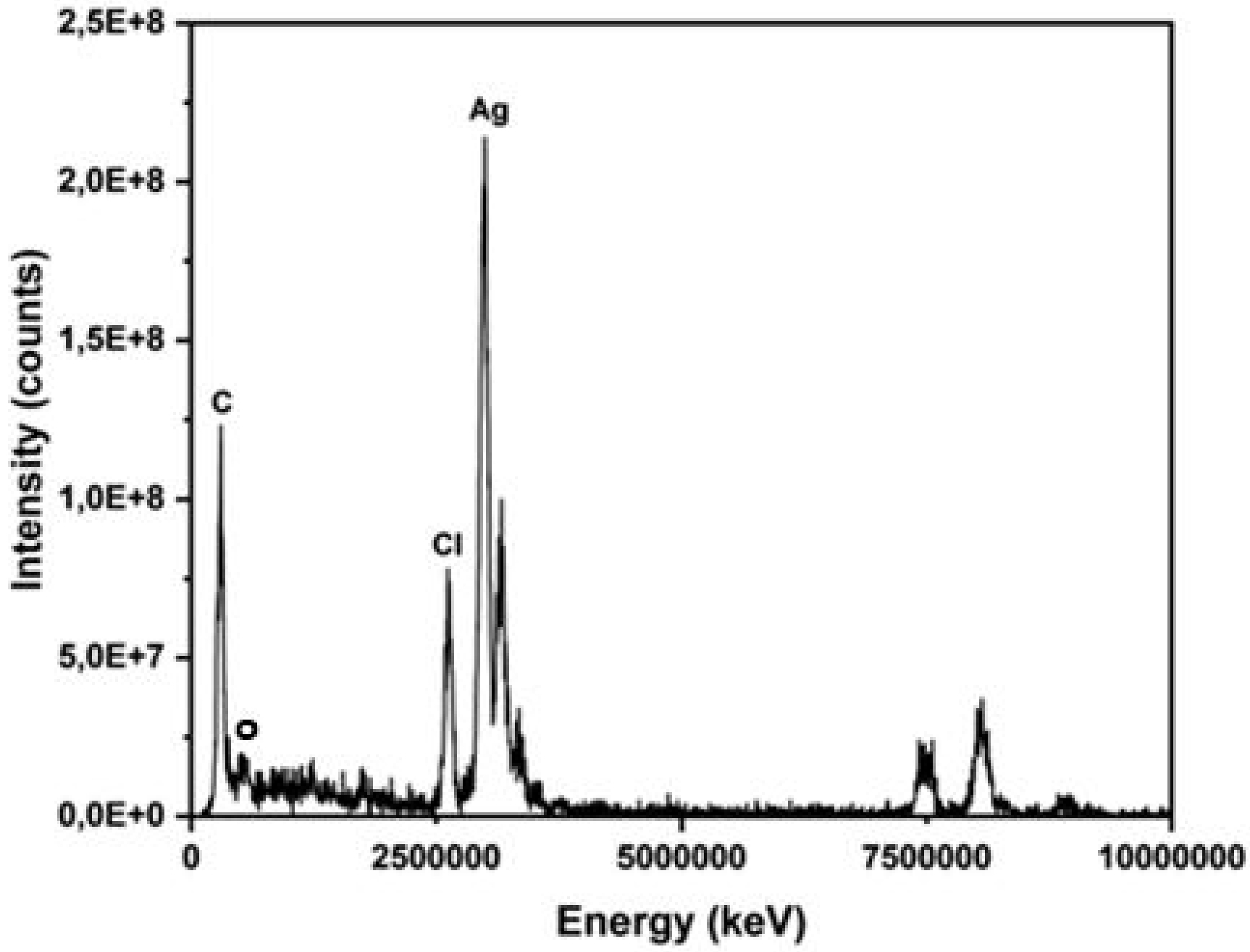

Figure S9. Energy dispersive spectroscopy (EDS) spectrum reveals the components (Ag, Cl, C, and O) of TbEst@Ag/AgCl-NPs.
